# Supplementary material for: Methionine aminopeptidase 2 and its autoproteolysis product have different binding sites on the ribosome
Source: Nat Commun. 2024 Jan 24;15:716. doi: 10.1038/s41467-024-44862-7 (PMC10808355; doi:10.1038/s41467-024-44862-7)
Supplement: Supplementary file 1 — Supplementary Information [file 41467_2024_44862_MOESM1_ESM.pdf]

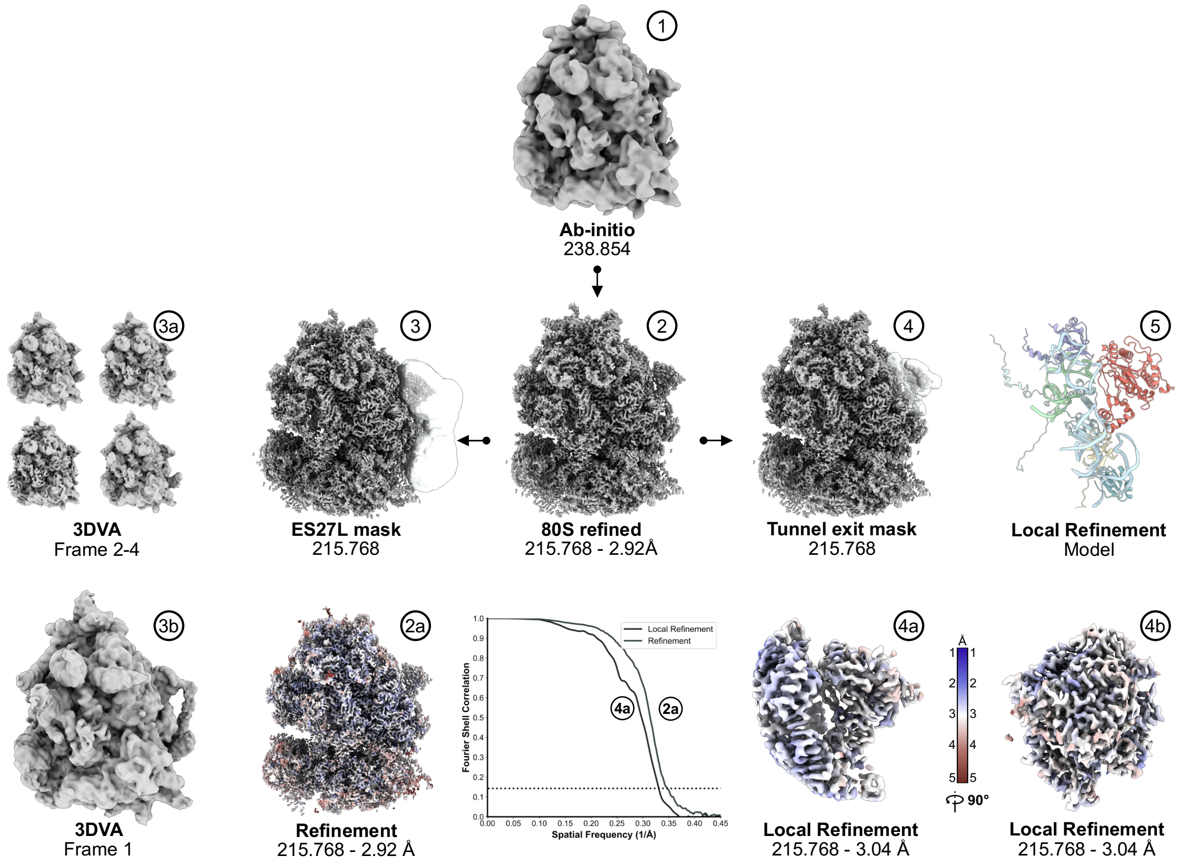

Supplementary Figure 1: Cryo-EM data processing of *Hs*MAP2 on *Hs*80S ribosomes using CryoSPARC. After pre-processing and three rounds of 2D classification, selected particles were subjected to *Ab-initio* reconstruction **(1)** to obtain a preliminary 3D volume. After further classification using Heterogeneous refinement, residual particles were used to perform Homogenous refinement, yielding a refined map of 2.92 Å resolution **(2)**. Masks were placed around the tunnel exit and ES27L **(3)** to perform focused 3D variability analysis. The output of this job was passed through a 3D variability display job into 5 particle clusters. All clusters were individually subjected to *Ab-initio* reconstruction, followed by Homogenous refinement. In one of the particle subsets, the refined map showed the position of ES27L **(3b)**, while the others lacked this feature **(3a)**. Using a mask around MAP2, a local refinement was performed **(4)**, yielding a local resolution of 3.04 Å **(4a,b)**. Local resolution estimations were performed on the overall refinement **(2a)**, as well as the local refinement **(4a)**. Additionally, FSC curves were plotted for these two refinements. Both refinements were used to build a model of MAP2 at the ribosomal tunnel exit **(5)**.

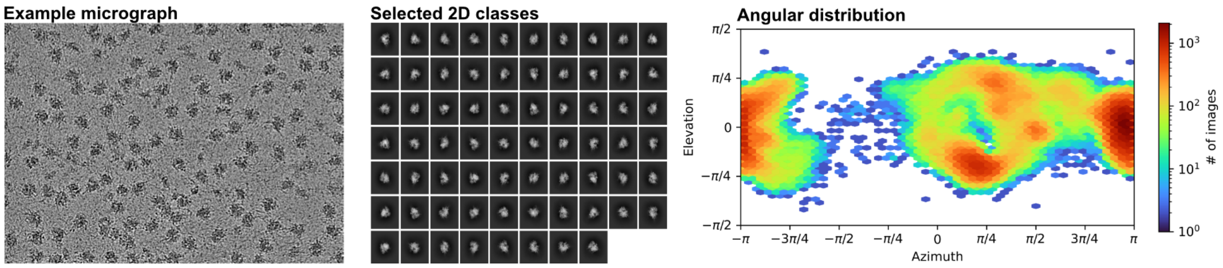

Supplementary Figure 2: Example micrograph, selected 2D classes and angular distribution of the *Hs*MAP2 dataset. Micrograph is shown at 84,000x magnification. All 2D classes that were used for the *Ab-initio* reconstruction are shown. Angular distributions are shown for the final refined map that was used for model building (Supplementary Fig. 1(2a)).

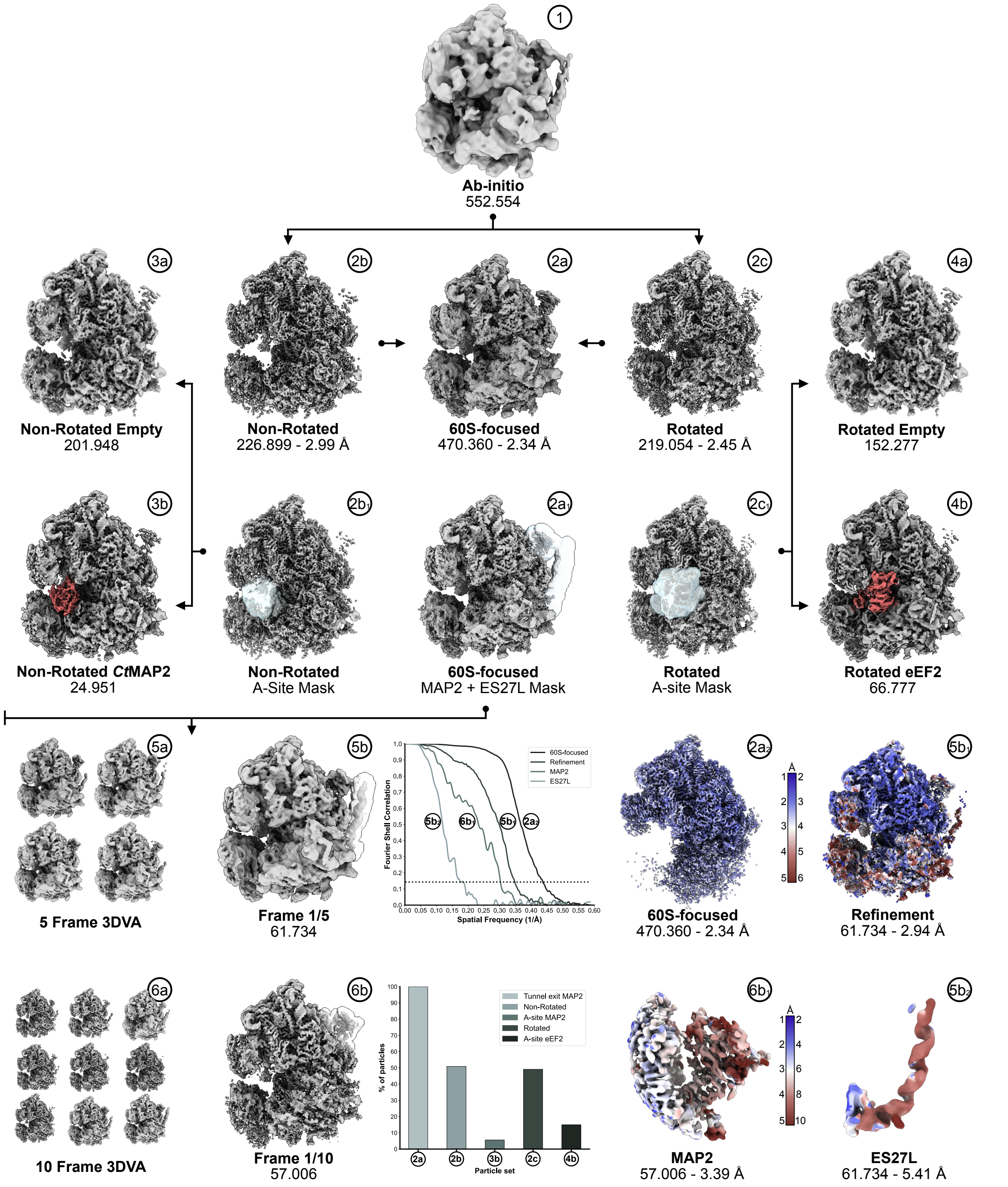

Supplementary Figure 3: Cryo-EM data processing of *Ct*MAP2 on *Ct80S* ribosomes using CryoSPARC. After pre-processing and 3 rounds of 2D classification, selected particles were subjected to *Ab-initio* reconstruction **(1)** to obtain a preliminary 3D volume. Heterogeneous refinement revealed that the 40S subunit was rotated in nearly half of all 80S particles. Particles in the rotated and non-rotated state were individually subjected to Homogenous refinement to obtain resolutions of 2.45 Å **(2c)** and 2.99 Å **(2b)** respectively. A mask was placed in the A-site **(2b1, c1)**, to perform local classification using 3D variability analysis. Particles were subsequently output into two clusters, along variability component 1, and subjected to *Ab-initio* reconstruction and Homogenous refinement. In ~11% (24,951 particles) of all particles

in the non-rotated state, MAP2 could be identified in the A-site **(3b)**, while the A-site was unoccupied for the remaining particles **(3a)**. The same procedure was performed for the A-site of 80S particles in the rotated state. Here, ~30% of ribosomes carried eEF2 **(4b)**, while the A-site was unoccupied for the remaining particles **(4a)**. Since the rotation of the 40S subunit had no apparent effect on the MAP2 interaction at the tunnel exit, particles from both subsets were merged and subjected to another Homogenous refinement **(2a)** reaching a resolution of 2.34 Å at the 60S subunit **(2a)**. Focused 3D variability analysis was performed with a mask around ES27L and MAP2, and particles were output into 5 and 10 clusters. Each particle cluster was subjected to *Ab-initio* reconstruction and Homogenous refinement. In cluster 1/5 **(5b)** ES27L was best defined, and a mask was placed around ES27L for a subsequent local refinement **(5b2)**. Likewise, cluster 1/10 **(6b)** revealed the highest resolution MAP2 density. A mask was placed around MAP2 and a local refinement was performed **(6b1)**. Local resolution estimations were performed for all maps that were used for model building **(2a1, 5b1, 5b2, 6b1)**, and FSC curves are plotted. The final model was refined into map 5b1. The abundance of particles in the rotated and non-rotated state, as well as the occupancy of the A-site and tunnel exit, are shown in a bar plot.

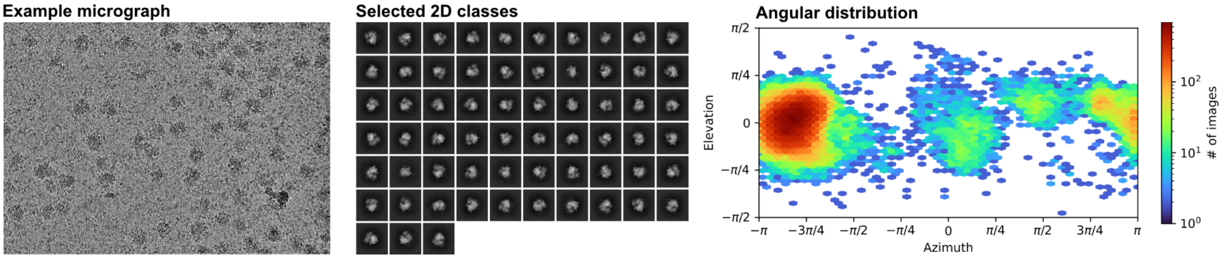

Supplementary Figure 4: Example micrograph, selected 2D classes and angular distribution of the *Ct*MAP2 dataset. Micrograph is shown at 105,000x magnification. All 2D classes that were used for the *Ab-initio* reconstruction are shown. Angular distributions are shown for the final refined map, that was used for model building (Supplementary Fig. 3(5b$_1$)).

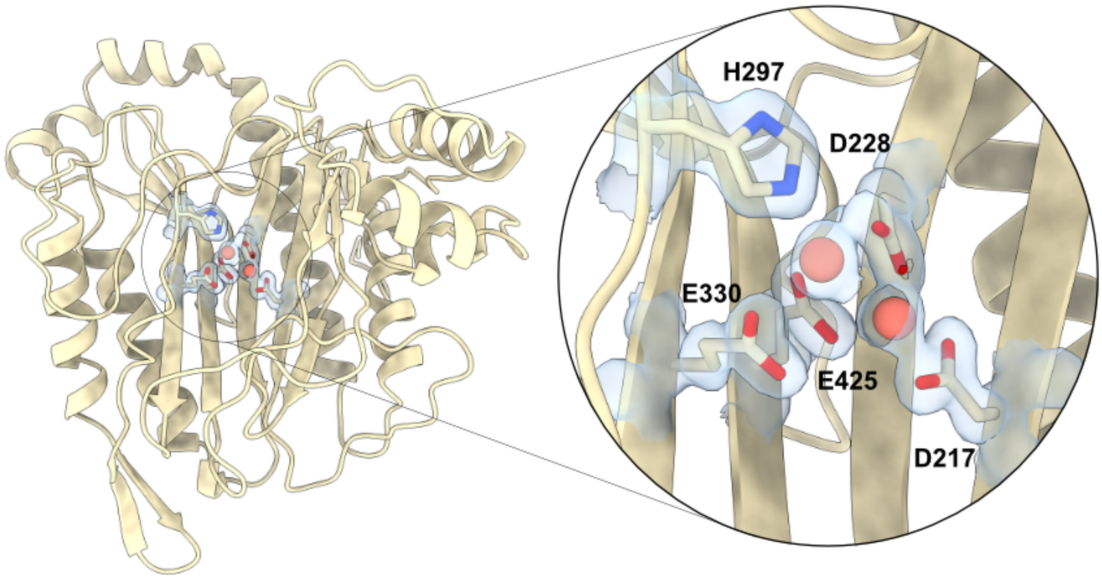

Supplementary Figure 5: Crystal structure of *Ct*MAP2ΔN at 1.3 Å resolution. The inset shows the active site with the five conserved catalytic residues. Two metal co-factors (red spheres) are coordinated by these residues. The $2mF_o$-DF_c density is shown around the catalytic residues and the metal cations (1.2σ level).

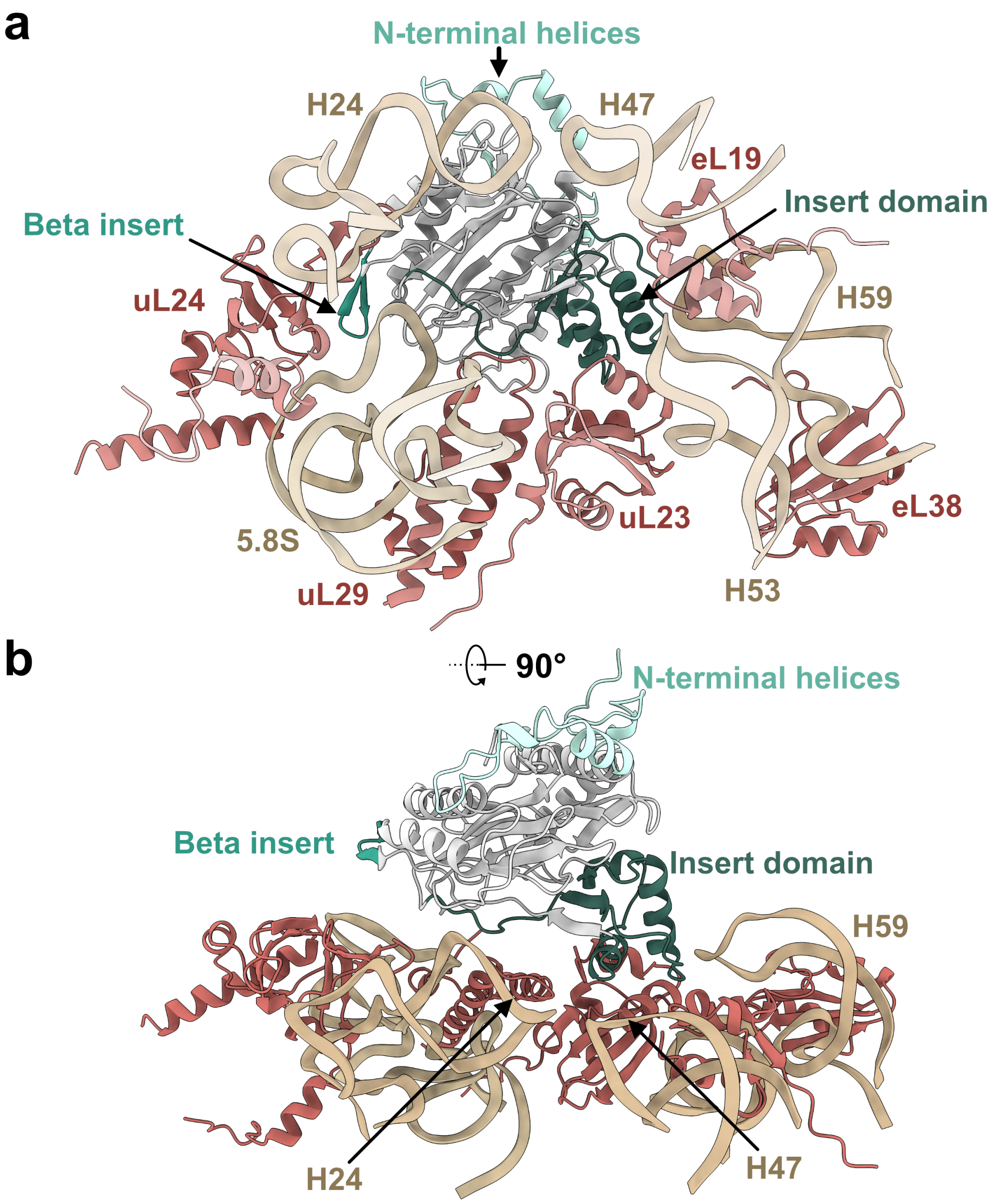

Supplementary Figure 6: Binding mode of MAP2 on the ribosomal tunnel exit. **(a)** View from inside the tunnel exit into the active site of *Ct*MAP2. The three main features that distinguish MAP2 from the plain pita-bread fold of MAP1 are highlighted in green. The Insert domain forms the main contact at the tunnel exit, while the Beta insert and N-terminal helices do not contribute to tunnel interaction. **(b)** Side view on the MAP2-80S interaction. The MAP2 vestibule forms centrally on the tunnel exit and is emphasized by a half circle.

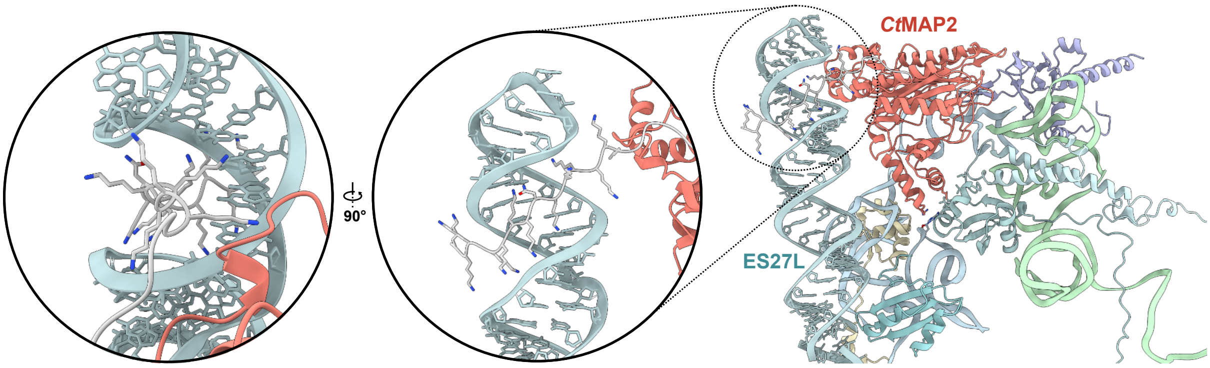

Supplementary Figure 7: Model of interaction between ES27L and the *Ct*MAP2-specific N-terminal extension. By superimposing an Alphafold prediction of *Ct*MAP2 onto our cryoEM structure of *Ct*MAP2/80S, the positively charged region of the N-terminal extension passes through the widened major groove of ES27L. This model is consistent with the interaction of stretches of positively charged residues within RAFs with widened RNA major grooves[1,2].

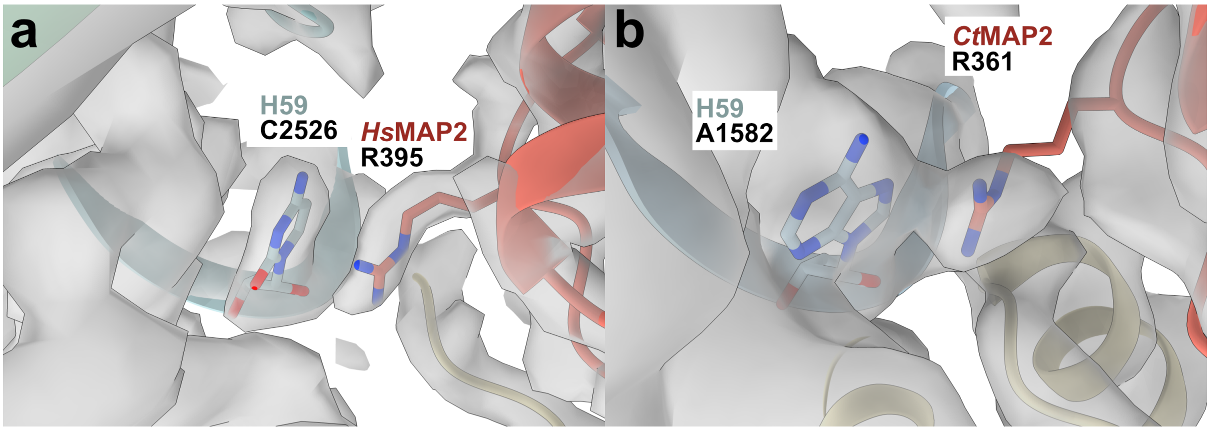

Supplementary Figure 8: Interaction of *Hs*MAP2-R395 **(a)** and *Ct*MAP2-R361 **(b)** with the PTE. Respective cryoEM maps are shown in grey. The guanidine group of the pivot-point arginine stacks onto C2526 and A1582 for *Hs*80S and *Ct*80S ribosomes, respectively.

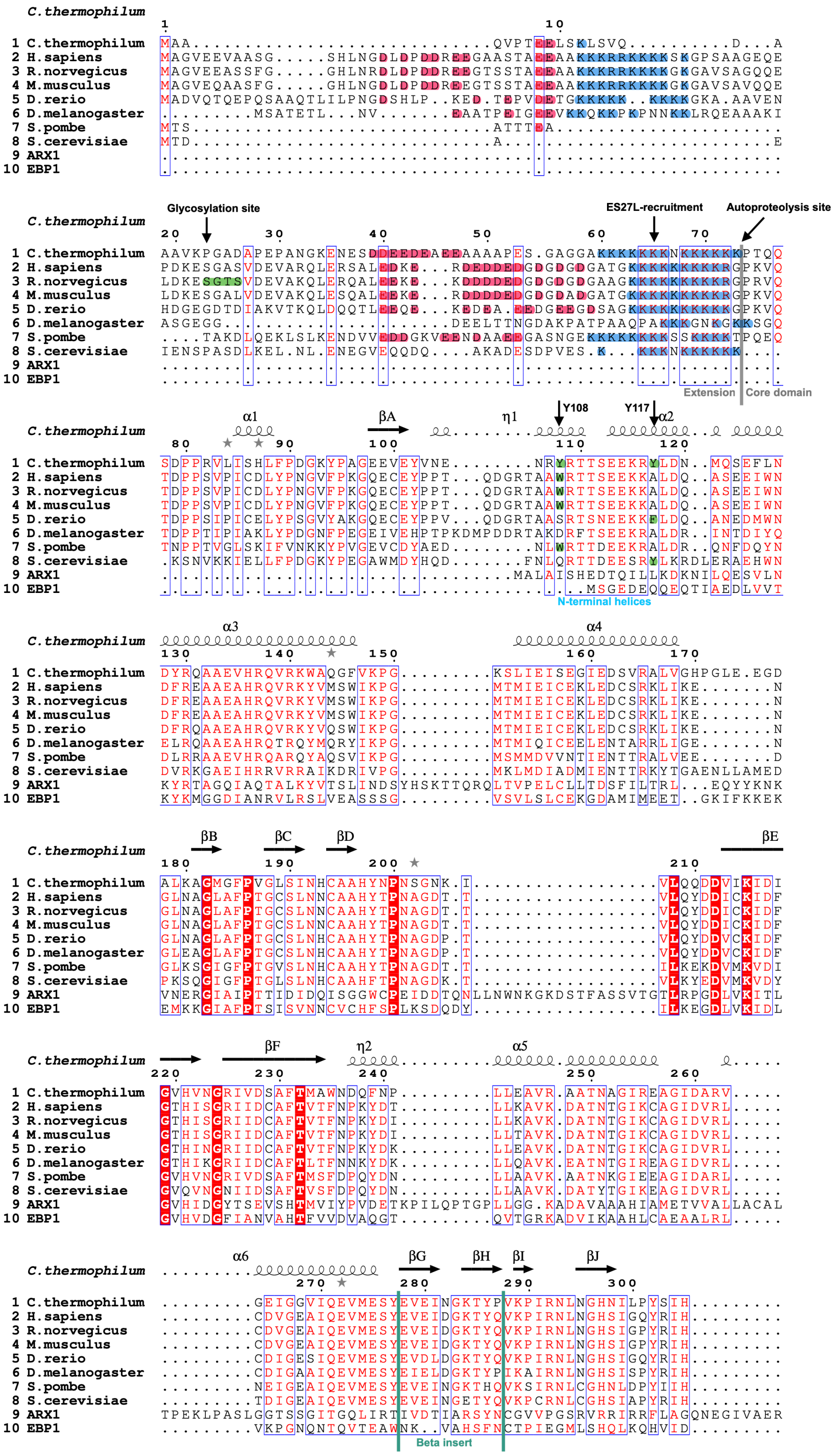
C.thermophilum
H.sapiens
R.norvegicus
M.musculus
D.rerio
D.melanogaster
S.pombe
S.cerevisiae
ARX1
EBP1
Glycosylation site
ES27L-recruitment
Autoproteolysis site
Extension
Core domain
Y108
Y117
N-terminal helices
Beta insert

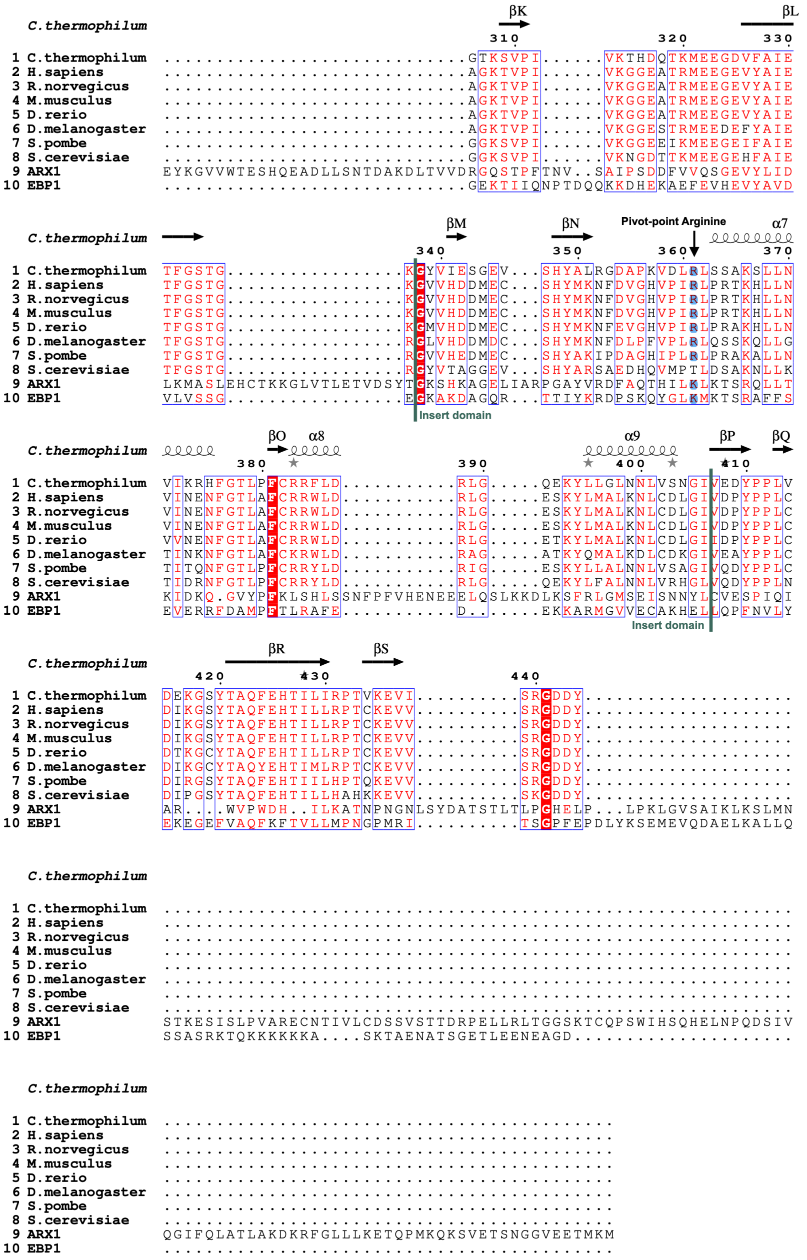

Supplementary Figure 9: Multiple sequence alignment of MAP2 from 8 different organisms, as well as MAP2-like proteins *Sc*Arx1 and *Hs*Ebp1. Secondary structure elements are shown for *Ct*MAP2 above the sequences, as present in the crystal structure. Conserved residues are highlighted in red. Poly-ionic regions at the N-terminus of MAP2 are highlighted in blue (cationic) and pink (anionic). The cationic region required for ES27L recruitment is indicated by an arrow, as is the predicted autoproteolysis site. The glycosylation site at the N-terminus of rat *Rn*MAP2 (ref. 3) is highlighted in green. Important residues for A-site binding of *Ct*MAP2 (Y108 and Y117) are also indicated by arrows. The conserved arginine that serves as the pivot point for MAP2 rotation is highlighted in blue.

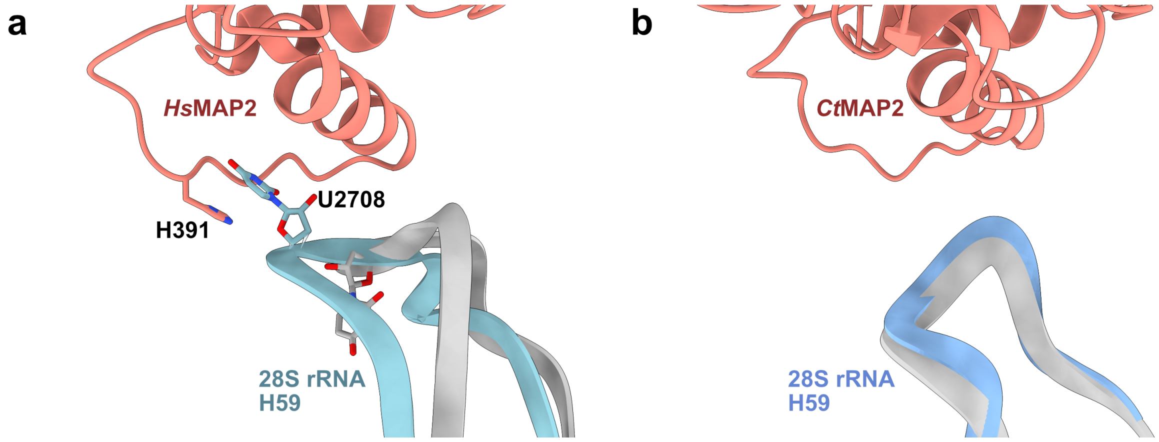

Supplementary Figure 10: Effects of MAP2 binding on the conformation of H59. **(a)** Binding of the *Hs*MAP2 insert domain causes a structural remodeling of H59. The remodeled rRNA helix is shown in teal, the idle conformation in the absence of MAP2 is superimposed in grey[4]. **(b)** This remodeling does not take place on *Ct* ribosomes. The MAP2-bound conformation is shown in blue, the idle conformation is superimposed in grey[5].

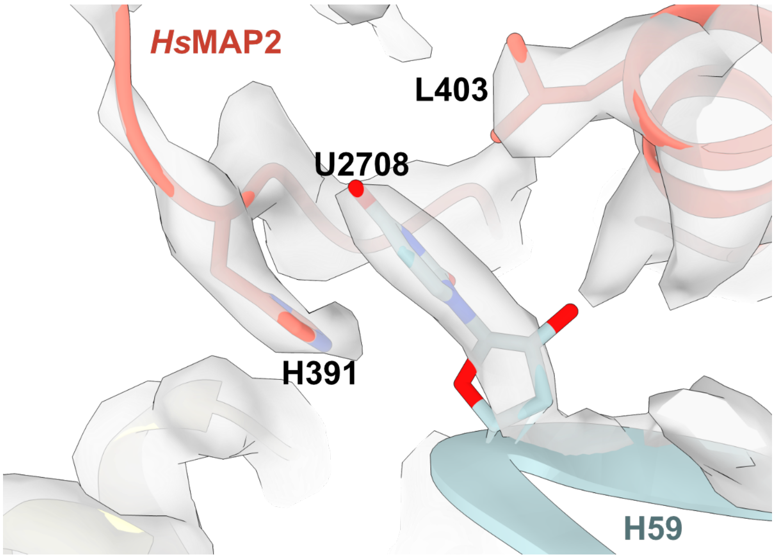

Supplementary Figure 11: Binding of *Hs*MAP2 causes a structural remodeling of H59 and U2708 pierces into the insert domain where it stacks in between H391 and L403 of *Hs*MAP2. The cryoEM map is shown in grey and residues of interest are labeled.

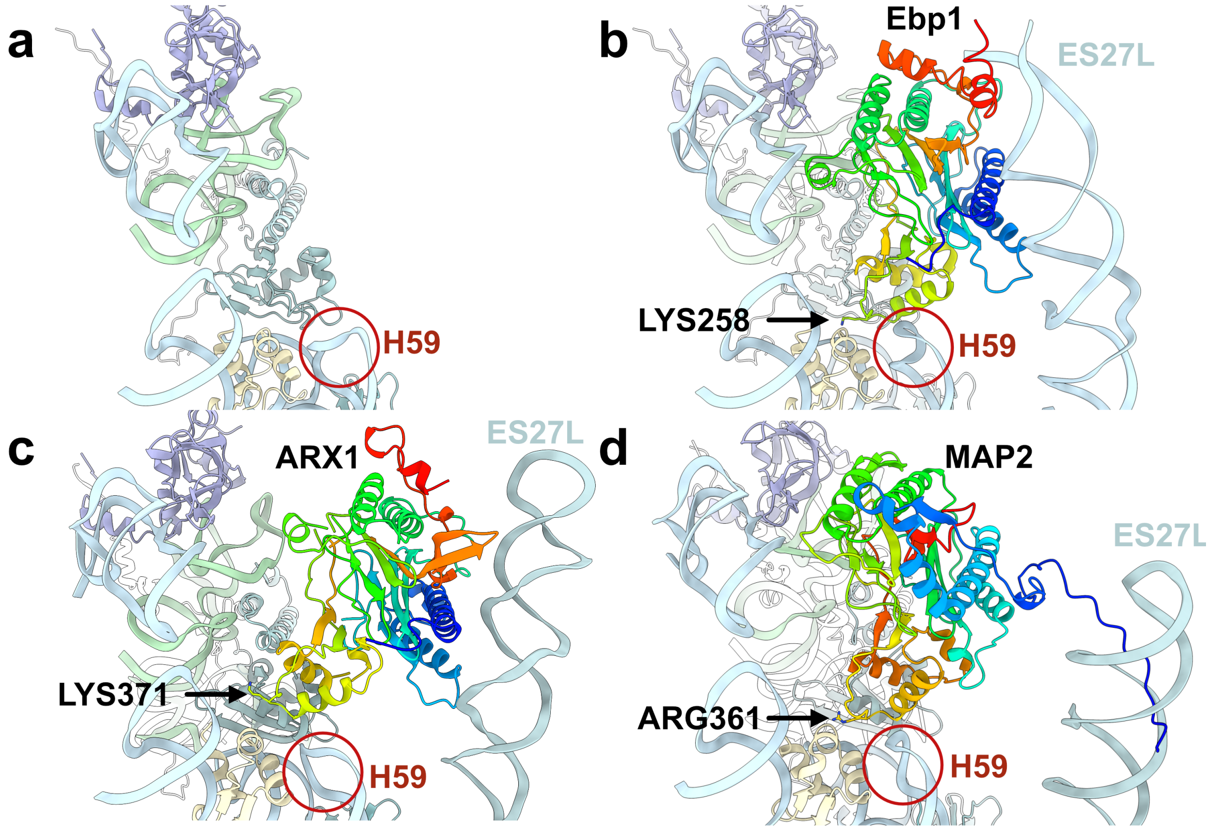

Supplementary Figure 12: Comparison of the ribosome binding mode between *Hs*Ebp1 (ref. 6), *Sc*Arx1 (ref. 7) and *Ct*MAP2. The proteins of interest are colored from blue (N-terminus) to red (C-terminus). **(a)** Structure of H59 at the PTE of *Hs*80S ribosomes in the absence of a bound MAP2-like protein[4]. **(b)** *Hs*Ebp1 binding induces a remodeling of H59. Unlike MAP2 and Arx1, Ebp1 contacts ES27L with both its N-terminus and C-terminus. In Ebp1 the pivot point arginine conserved in catalytically active MAP2s is absent, instead a lysine (Lys258) is at the corresponding position. **(c)** *Sc*Arx1 contacts ES27L with its N-terminus, similar to Ebp1, but does not show a second contact with the expansion segment. The arginine conserved in catalytically active MAP2s is replaced by a lysine (Lys371). **(d)** MAP2 binding is highly dynamic and also recruits ES27L. Unlike Ebp1, MAP2 only binds ES27L with the unstructured N-terminal extension (interaction derived from an Alphafold model, Supplementary Fig. 7). In contrast to *Hs*Ebp1 and *Hs*MAP2, *Ct*MAP2 does not remodel H59 (Supplementary Fig. 10).

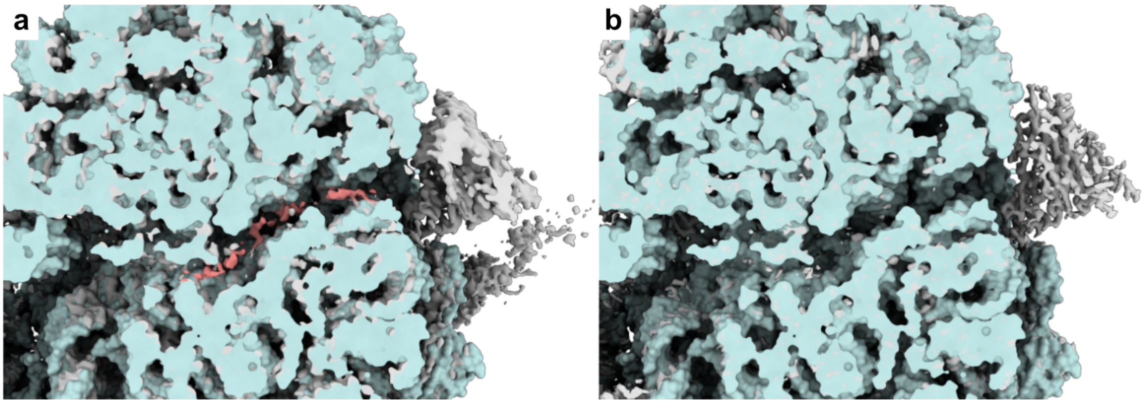

Supplementary Figure 13: Cross sections of cryo-EM maps for *Ct* **(a)** and human ribosomes **(b)** (light blue) in complex with MAP2 (grey). The nascent chain (red) is only visible in the exit tunnel of *Ct* ribosomes.

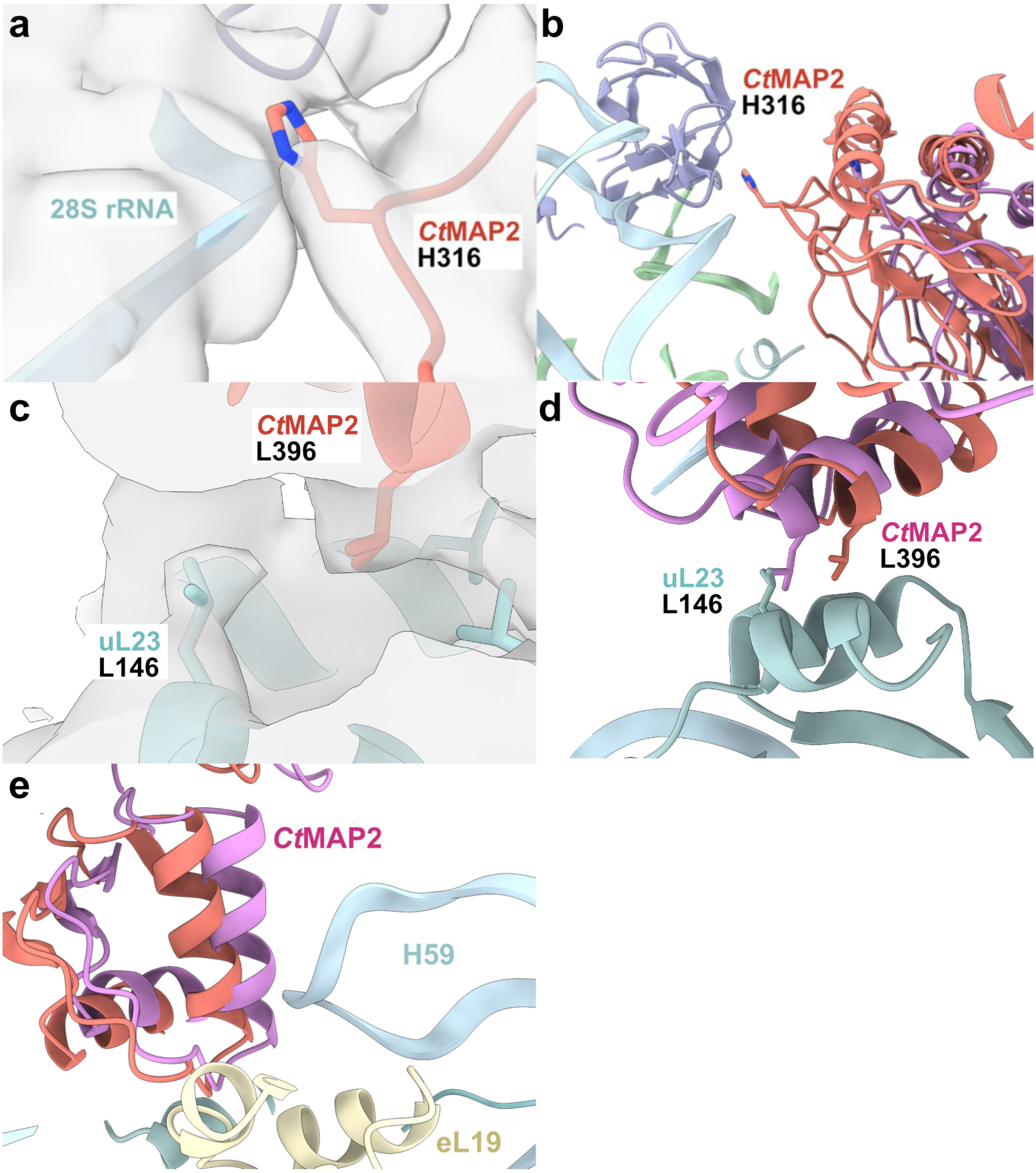

Supplementary Figure 14: *Ct*MAP2 rotation is limited by several constraints. Cryo-EM maps are shown in grey. MAP2 is shown in red when closest to the ribosome, and shown in purple when fully rotated away from the PTE. **(a** and **b)** *Ct*MAP2-H316 interacts with the 28S rRNA. This interaction is weakened as MAP2 rotates away from the tunnel exit. **(c** and **d)** *Ct*MAP2 moves ~5 Å along the surface of uL23 as MAP2 rotates away. The two leucine residues would clash if MAP2 would move further along this trajectory. **(e)** *Ct*MAP2 rotation moves the longest helix of the insert domain closer to the protruding 28S rRNA H59.

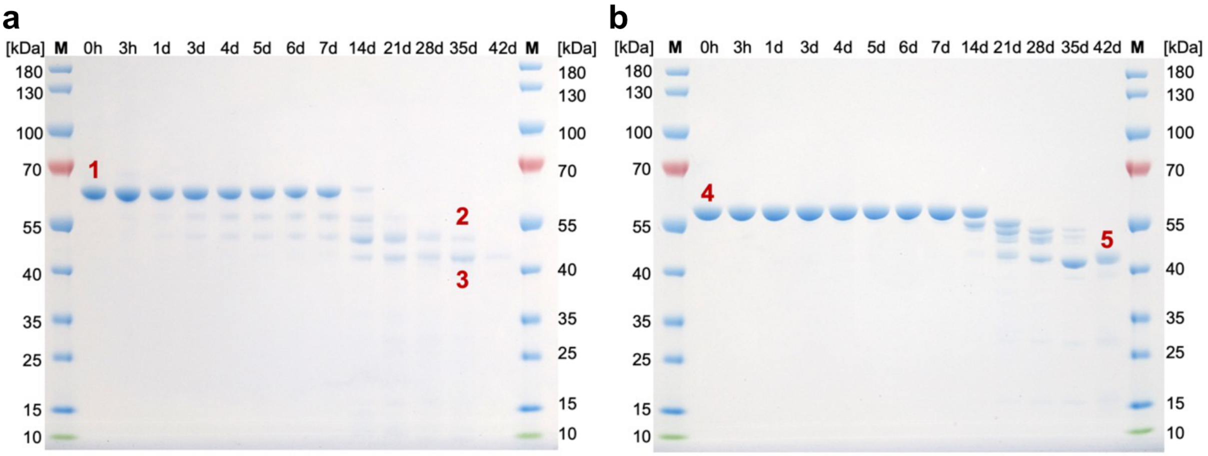

Supplementary Figure 15: Degradation of *Hs*MAP2 **(a)** and *Ct*MAP2 **(b)** at 4°C. MAP2 was purified from insect cells and stored at 4°C at a concentration of 1 mg/ml. Samples were collected at different intervals and analyzed by SDS-PAGE. **(a)** Full length *Hs*MAP2 (band 1) starts degrading after 3 days and yields two bands after 35 days (bands 2 and 3). Band 3 corresponds to the core domain at 41.5 kDa. **(b)** *Ct*MAP2 shows enhanced stability and only starts degrading after 14 days. *Ct*MAP2 degrades into a single stable band at 41.3 kDa, corresponding to the core domain.

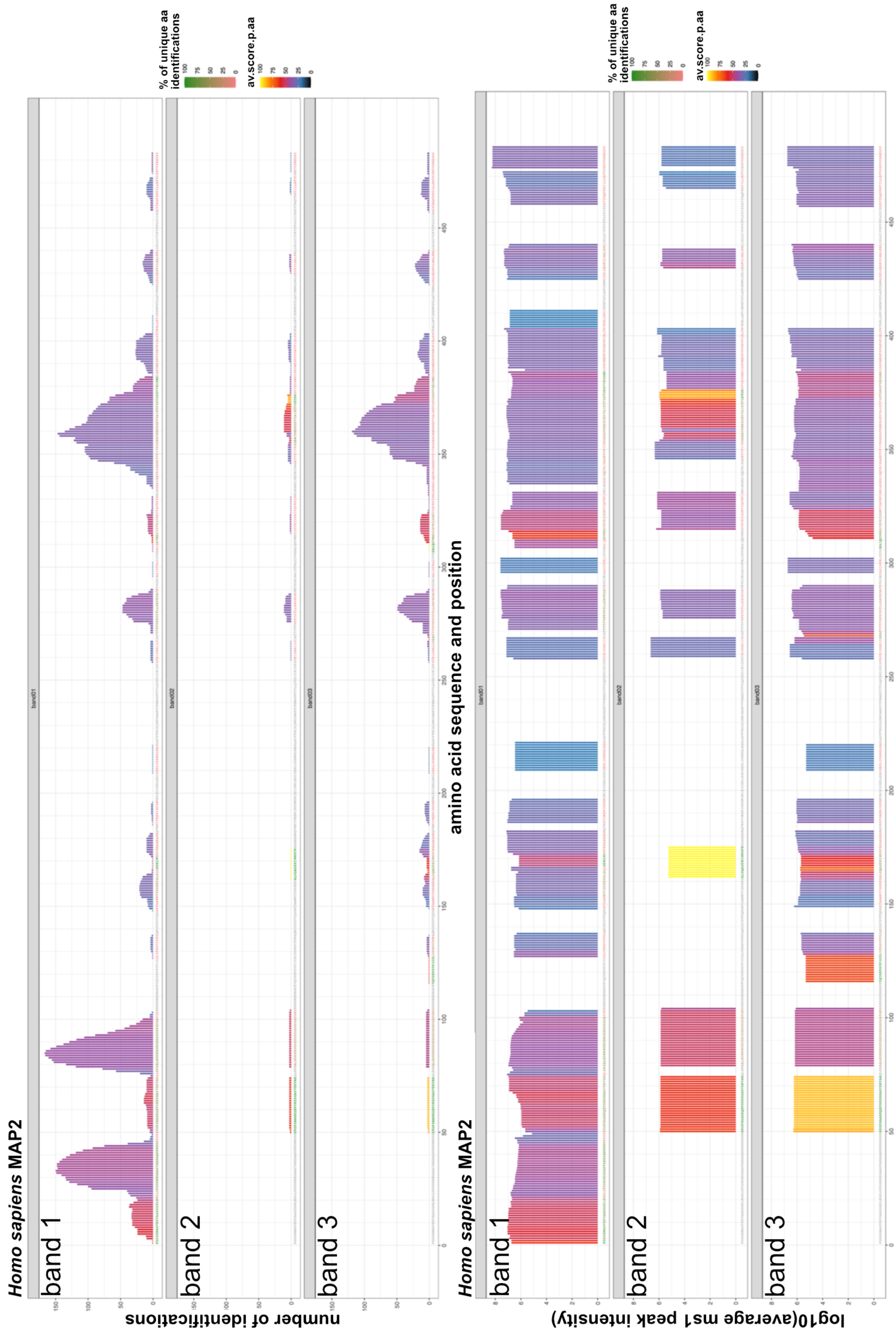

Supplementary Figure 16: Mass Spectrometry analysis of *Hs*MAP2 autoproteolysis. Bands 1, 2 and 3 (Supplementary Fig. 15) were analyzed by acid hydrolysis. Band 1 corresponds to full length protein and signal of the N-terminal residues can be detected. Analysis of the autoproteolysis products (band 2 and 3) confirm that the N-terminal residues are affected, while the C-terminus is still intact.

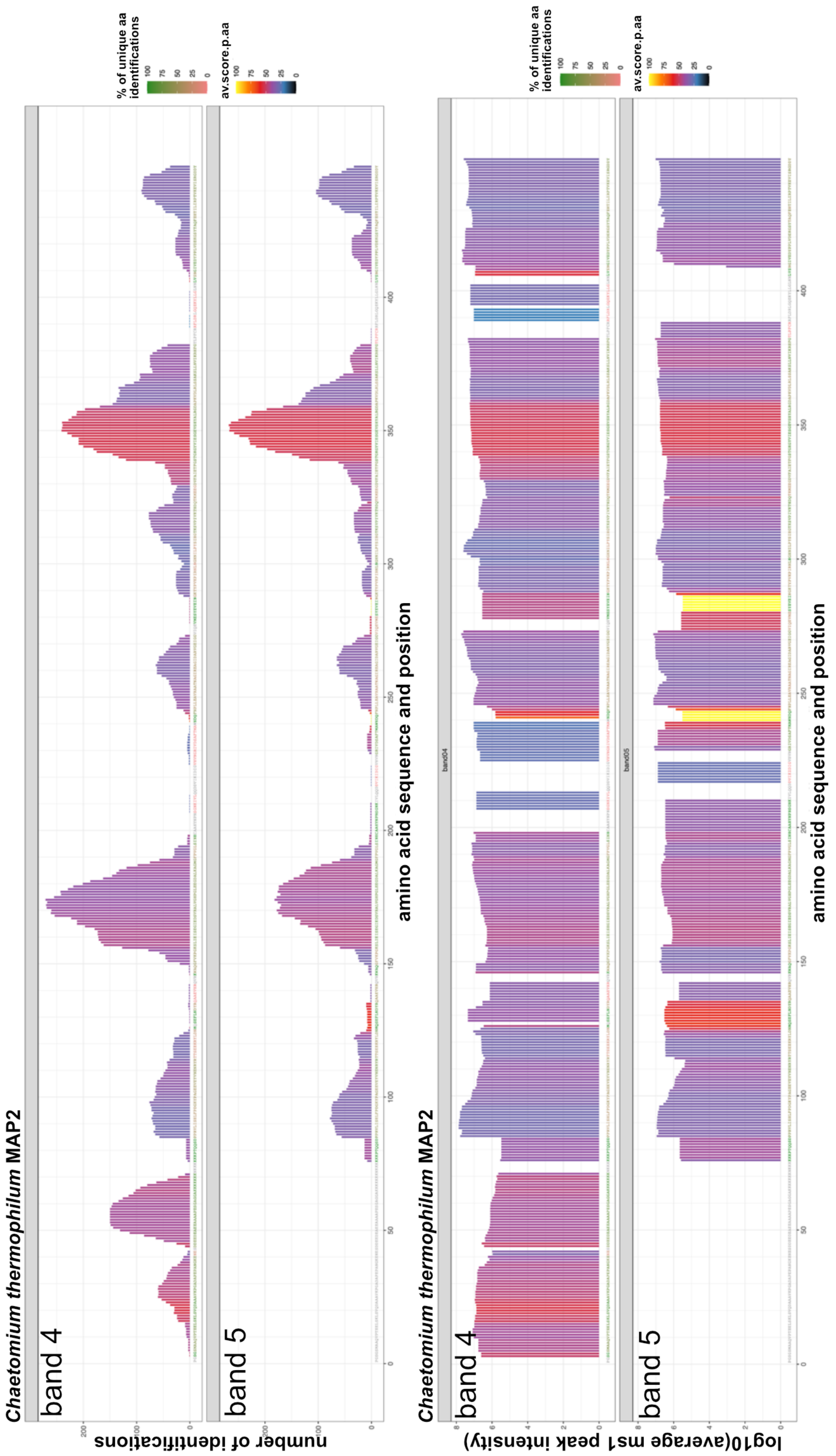

Supplementary Figure 17: Mass Spectrometry analysis of *Ct*MAP2 autoproteolysis. Bands 4 and 5 (Supplementary Fig. 15) were analyzed by acid hydrolysis. Band 4 corresponds to full length protein and signal of the N-terminal residues can be detected. Analysis of the autoproteolysis product (band 5) confirms that the N-terminal residues are affected, while the C-terminus is still intact.

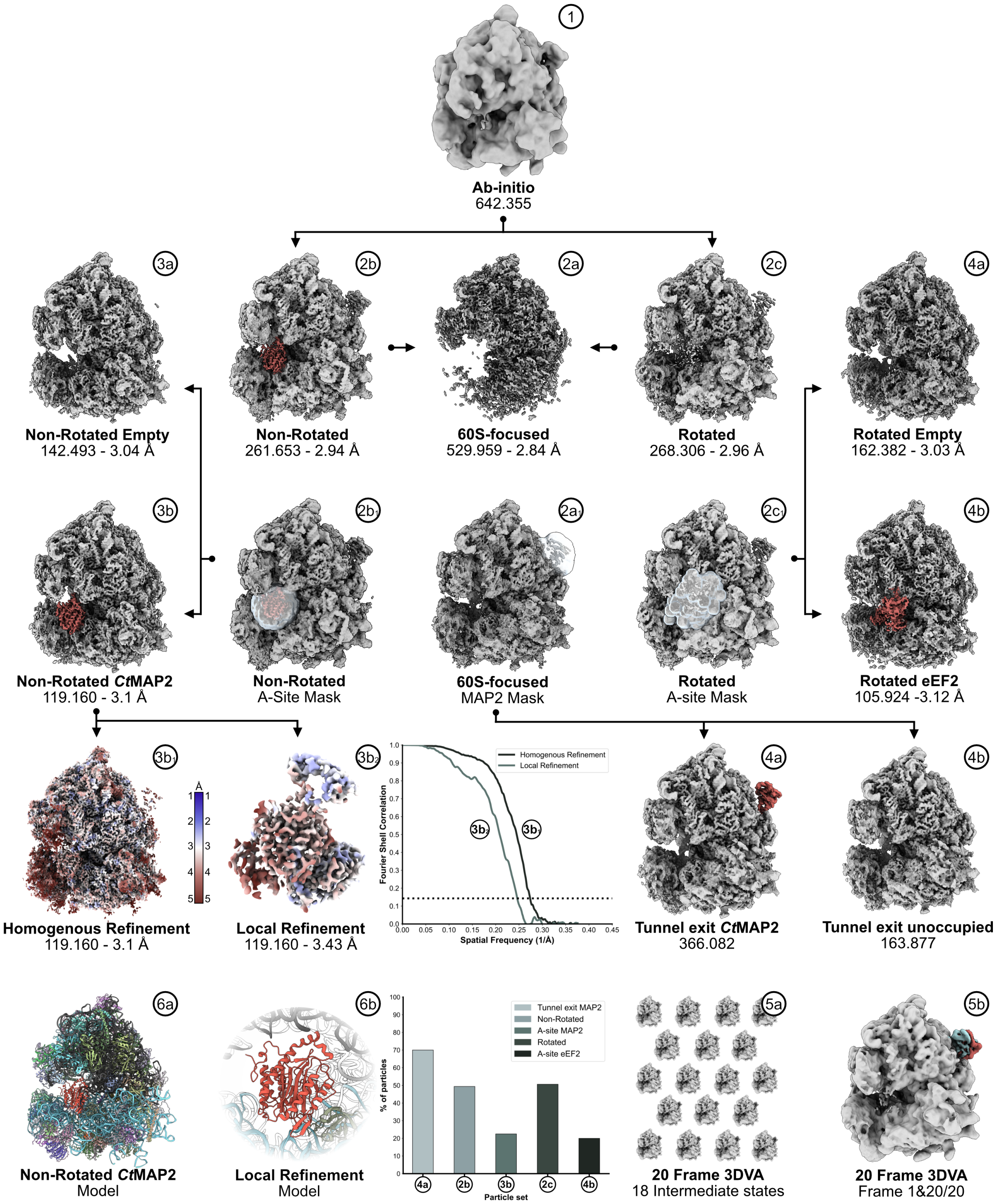

Supplementary Figure 18: Cryo-EM data processing of *Ct*MAP2ΔN on *Ct80S* ribosomes using CryoSPARC. After pre-processing and 3 rounds of 2D classification, selected particles were subjected *to Ab-initio* reconstruction **(1)** to obtain a preliminary 3D volume. Heterogeneous refinement revealed that the 40S subunit was rotated in nearly half of all 80S particles. Particles in the rotated and non-rotated state were individually subjected to Homogenous refinement to obtain resolutions of 2.96 Å **(2c)** and 2.94 Å **(2b)**, respectively. A mask was placed in the A-site **(2b1, c1)**, to perform local classification using 3D variability analysis. Particles were subsequently output

into two clusters, along variability component 1, and subjected to *Ab-initio* reconstruction and Homogenous refinement. In ~46% (119,160 particles) of all particles in the non-rotated state, *Ct*MAP2ΔN could be identified in the A-site **(3b)**, while the A-site was unoccupied for the remaining particles **(3a)**. The same procedure was performed for the A-site of 80S particles in the rotated state. Here, ~39% of ribosomes carried eEF2 **(4b)**, while the A-site was unoccupied for the remaining particles **(4a)**. Since the rotation of the 40S subunit had no apparent effect on the *Ct*MAP2ΔN interaction at the tunnel exit, particles in the rotated and non-rotated state were merged and subjected to another Homogenous refinement **(2a)** reaching a resolution of 2.84 Å at the 60S subunit **(2a)**. Focused 3D variability analysis was performed with a mask around *Ct*MAP2ΔN, and particles were output into 2 and 20 clusters. When outputting particles into 2 clusters along variability component 1, particles that had *Cz*MAP2ΔN on the tunnel exit **(4a)** could be separated from those where the tunnel exit was unoccupied **(4b)**. 3D variability analysis into 20 clusters **(5a,b)** revealed that *Ct*MAP2ΔN undergoes a rotation at the tunnel exit **(5b)**. Using the particles that had *Ct*MAP2ΔN in the A-site **(3b)**, a local refinement was performed. Subsequently, Local resolution estimations were performed for the overall refinement and the local refinement **(3b, b2)**, which were used for model building **(6a, b)**. The abundance of particles in the rotated and non-rotated state, as well as the occupancy of the A-site and tunnel exit are shown in a bar plot. FSC curves are plotted for both maps that were used for model building.

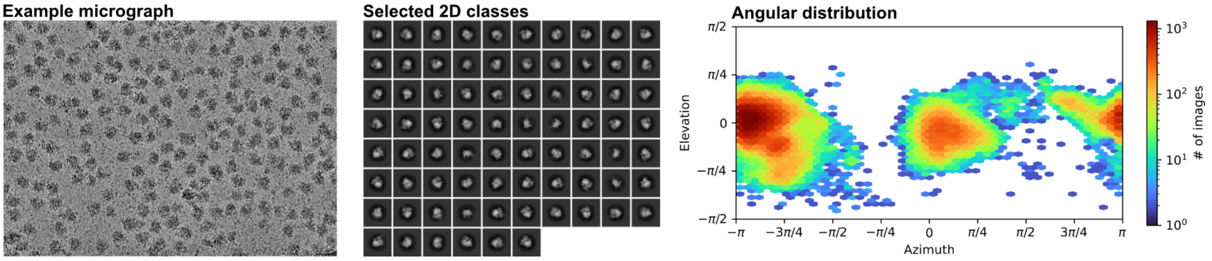

Supplementary Figure 19: Example micrograph, selected 2D classes and angular distribution of the *Ct*MAP2ΔN dataset. Micrograph is shown at 84,000x magnification. All 2D classes that were used for the *Ab-initio* reconstruction are shown. Angular distributions are shown for the final refined map, that was used for model building (Supplementary Fig. 18($3b_1$)).

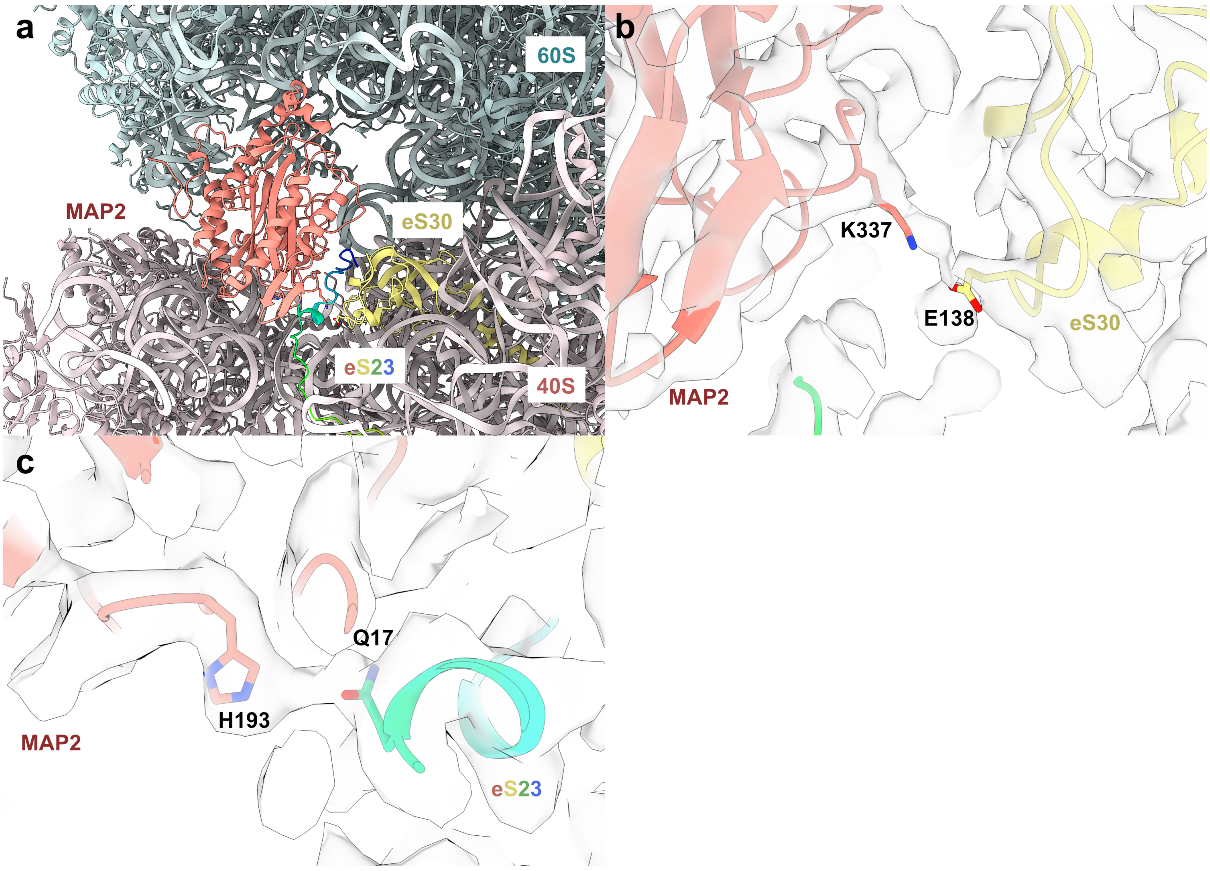

Supplementary Figure 20: *Ct*MAP2ΔN binding in the A-site. MAP2ΔN contacts 40S subunit protein eS30 (yellow) **(a)**. In the presence of MAP2ΔN, the N-terminus of eS23 (rainbow) becomes ordered. MAP2ΔN engages in polar and charged interactions with eS30 **(b)** and the N-terminus of eS23 **(c)**. The cryo-EM map is shown in grey.

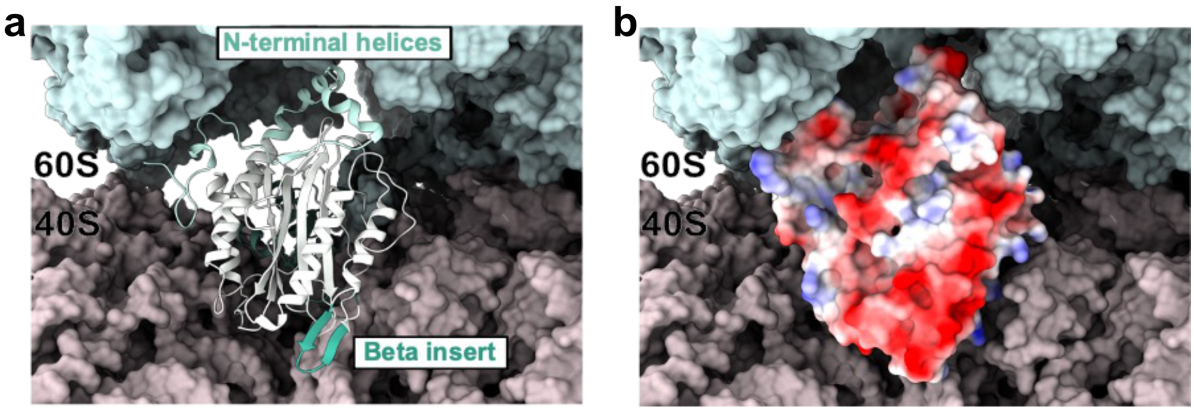

Supplementary Figure 21: Overall binding mode of *Ct*MAP2ΔN in the ribosomal A-site. **(a)** Front view onto the A-site position of the ribosome. *Ct*MAP2ΔN binds in the interface between the 40S and 60S subunit. The N-terminal helices interact with the 60S subunit. The Beta insert is not involved in binding. **(b)** The negatively charged Beta insert is solvent exposed and faces the entry of the ribosomal factor binding site.

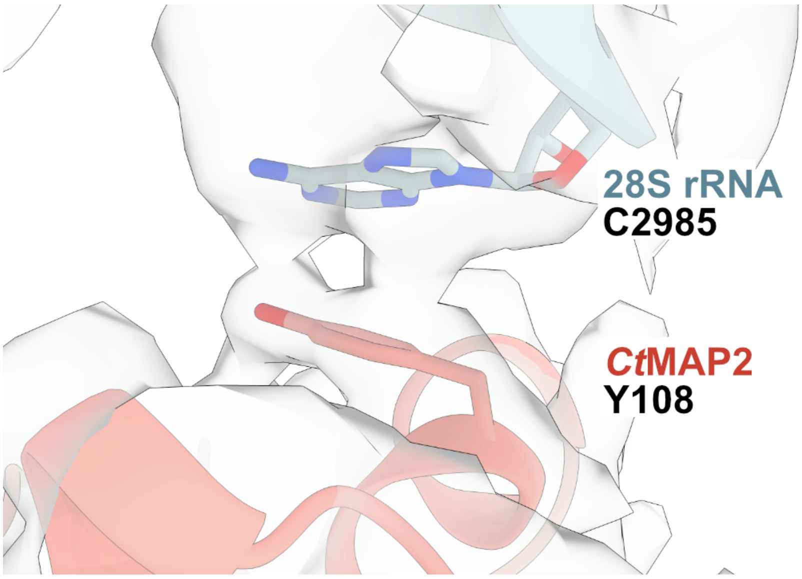

Supplementary Figure 22: Details of the 60S interaction of *Ct*MAP2ΔN bound to the A-site. Binding of *Ct*MAP2ΔN in the A-site stabilizes the N-terminal helices and involves π-stacking interactions (shown for Y108 and 28S rRNA C2985). The cryo-EM map is shown in grey.

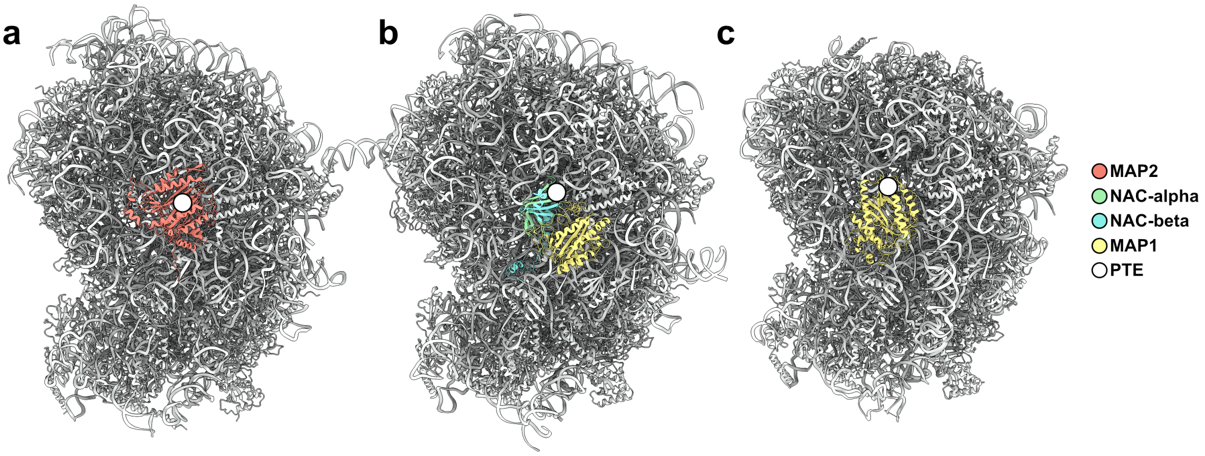

Supplementary Figure 23: Comparison of MAP2 and MAP1 binding at the PTE. **(a)** MAP2 sits centrally on the PTE and its active site faces the ribosome (this study). **(b)** Mammalian MAP1 binding is mediated by NAC-beta. MAP1 is positioned off-center from the PTE[8]. **(c)** Yeast MAP1 can bind the ribosome independent of NAC, and in a different position. Its binding mode differs from MAP2 and its active site is further away from emerging nascent chains[9].

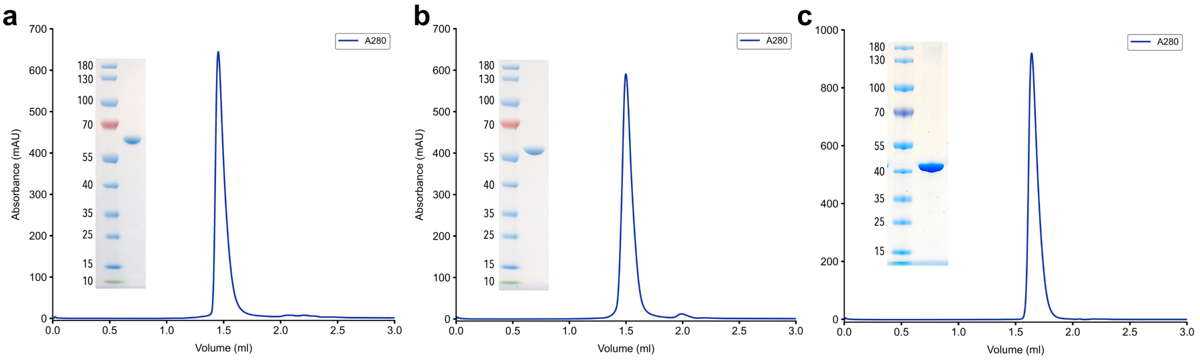

Supplementary Figure 24: Analytical SEC and SDS-gels of purified proteins used for cryo-EM sample preparation. SEC runs were performed on a S200/3.2-300 Increase column. **(a)** Purified *Hs*MAP2, **(b)** *Ct*MAP2, and **(c)** *Ct*MAP2ΔN. Molecular weights for the protein marker are given in kDa.

Supplementary Table 1: Cryo-EM data refinement statistics

**Model**	***hs*MAP2/80S**	***ct*MAP2/80S**	***ct*MAP2ΔN/80S**
Data collection statistics			
Microscope	Titan Krios	Titan Krios	Titan Krios
Camera	K2	K3	K2
Voltage (kV)	300	300	300
Magnification	84,000	105,000	84,000
Total dose (e^{-}/Å^{2})	42	42.7	54.8
Defocus range (μm)	-1.1 to -2.1	-1.1 to -2.1	-1.1 to -2.1
Calibrated pixel size (Å)	1.11	0.868	1.11
Refinement statistics			
Refined particles	215.768	61.734	119.160
Resolution (Å)	2.9	2.9	3.1
Chains	9	8	93
Atoms	12393 (Hydrogens: 0)	14990 (Hydrogens: 0)	208771 (Hydrogens: 0)
Residues	Protein: 960 Nucleotide: 214	Protein: 991 Nucleotide: 329	Protein: 12237 Nucleotide: 5230
Water	-	-	-
Ligands	Co: 2	-	ZNS: 3, B8N: 1, ZNL: 5
Bonds (RMSD*)			
Length (Å) (# > 4σ)	0.003 (0)	0.004 (0)	0.006 (7)
Angles (°) (# > 4 σ)	0.638 (1)	0.743 (13)	0.614 (89)
MolProbity score	1.34	1.62	1.68
Clash score	3.66	11.10	7.15
Ramachandran plot (%)			
Outliers	0.00	0.00	0.03
Allowed	3.06	2.25	4.17
Favored	96.94	97.75	95.80
Rama-Z (Ramachandran Plot, Z-score, RMSD*)			
whole (N = 951)	0.62 (0.25)	0.33 (0.25)	-1.04 (0.07)
helix (N = 379)	1.67 (0.26)	1.67 (0.25)	-0.14 (0.08)
sheet (N = 107)	1.60 (0.52)	0.62 (0.43)	-0.15 (0.12)
loop (N = 465)	-0.98 (0.24)	-0.09 (0.28)	-1.15 (0.07)
Rotamer outliers (%)	0.00	0.00	0.16
Cβ outliers (%)	0.00	0.00	0.00
Peptide plane (%)			
Cis proline/general	0.0/0.0	2.3/0.0	0.8/0.0
Twisted proline/general	0.0/0.0	0.0/0.0	0.2/0.0
CaBLAM outliers (%)	1.71	1.03	2.72
ADP (B-factors)			
Iso/Aniso (#)	12393/0	14990/0	208771/0
min/max/mean			
Protein	11.76/71.25/41.64	15.28/125.97/49.34	46.72/625.58/113.03
Nucleotide	18.95/124.23/44.59	24.96/235.84/10.57	47.08/689.64/103.76
Ligand	88.76/99.16/93.96	-	70.69/161.53/134.19
Occupancy (%)	100.00	100.00	100.00
Model vs. Data (CC mask)	0.84	0.77	0.87
Resolution according to model vs. map FSC = 0.143 (masked) (Å)	2.9	2.8	3.1

* RMSD: root-mean-squared-deviation

Supplementary Table 2: X-ray data collection and refinement statistics for *Ct*MAP2ΔN

Data collection	
Wavelength (Å)	0.9737
Resolution range (Å)	46.53 - 1.3 (1.32-1.3)
Space group	P 21 21 21
Unit cell (Å)	59.94 73.81 82.05 90 90 90
Total reflections	1103571 (35025)
Unique reflections	90066 (4362)
Multiplicity	12.3 (8.0)
Completeness (%)	99.9 (99.0)
Mean I/sigma (I)	23.0 (2.1)
Wilson B-factor (Å^2)	17.1
R_{pim}	0.015 (0.288)
CC1/2	0.999 (0.879)
Refinement	
Resolution range (Å)	40.47-1.3 (1.35-1.3)
Reflections used in refinement	89942 (8840)
R_{work}	0.129 (0.198)
R_{free}	0.156 (0.234)
Number of non-hydrogen atoms	3573
macromolecules	3043
ligands	20
solvent	522
Protein residues	371
RMS (bonds)	0.010
RMS (angles)	1.12
Ramachandran favored (%)	98.37
Ramachandran allowed (%)	1.63
Ramachandran outliers (%)	0.00
Rotamer outliers (%)	0.91
Clashscore	2.97
Average B-factor (Å^2)	24.43
macromolecules	22.39
ligands	36.16
solvent	36.15

Statistics for the highest-resolution shell are shown in parentheses.
R_{free} is for 5% of all data.

Supplementary References

1. Grotwinkel, J. T., Wild, K., Segnitz, B. & Sinning, I. SRP RNA remodeling by SRP68 explains its role in protein translocation. *Science* **344**, 101-104, (2014).
2. Kišonaitė, M. *et al.* Structural inventory of cotranslational protein folding by the eukaryotic RAC complex. *Nat Struct Mol Biol* **30**, 670-677, (2023).
3. Datta, R., Choudhury, P., Ghosh, A. & Datta, B. A glycosylation site, 60SGTS63, of p67 is required for its ability to regulate the phosphorylation and activity of eukaryotic initiation factor 2alpha. *Biochemistry* **42**, 5453-5460, (2003).
4. Natchiar, S. K., Myasnikov, A. G., Kratzat, H., Hazemann, I. & Klaholz, B. P. Visualization of chemical modifications in the human 80S ribosome structure. *Nature* **551**, 472-477, (2017).
5. Kišonaitė, M., Wild, K., Lapouge, K., Ruppert, T. & Sinning, I. High-resolution structures of a thermophilic eukaryotic 80S ribosome reveal atomistic details of translocation. *Nat Commun* **13**, 476, (2022).
6. Wild, K. *et al.* MetAP-like Ebp1 occupies the human ribosomal tunnel exit and recruits flexible rRNA expansion segments. *Nat Commun* **11**, 776, (2020).
7. Greber, B. J., Boehringer, D., Montellese, C. & Ban, N. Cryo-EM structures of Arx1 and maturation factors Rei1 and Jjj1 bound to the 60S ribosomal subunit. *Nat Struct Mol Biol* **19**, 1228-1233, (2012).
8. Gamerdinger, M. *et al.* NAC controls cotranslational N-terminal methionine excision in eukaryotes. *Science* **380**, 1238-1243, (2023).
9. Knorr, A. G. *et al.* The dynamic architecture of Map1- and NatB-ribosome complexes coordinates the sequential modifications of nascent polypeptide chains. *PLoS Biol* **21**, e3001995, (2023).
