# Supplementary material for: Methionine aminopeptidase 2 and its autoproteolysis product have different binding sites on the ribosome
Source: Nat Commun. 2024 Jan 24;15:716. doi: 10.1038/s41467-024-44862-7 (PMC10808355; doi:10.1038/s41467-024-44862-7)
Supplement: Supplementary file 3 — Description of Additional Supplementary Files [file 41467_2024_44862_MOESM3_ESM.pdf]

Description of Additional Supplementary Files:

Supplementary Data 1:

1) Primers.xlsx (primers referenced in the Sample preparation section)
2) MS_databank.fasta (database for Mass Spec. peptide identification generated In-house at the EMBL MS facility)
